# Supplementary material for: Mitoribosome structure with cofactors and modifications reveals mechanism of ligand binding and interactions with L1 stalk
Source: Nat Commun. 2024 May 20;15:4272. doi: 10.1038/s41467-024-48163-x (PMC11106087; doi:10.1038/s41467-024-48163-x)
Supplement: Supplementary file 1 — Supplementary Information [file 41467_2024_48163_MOESM1_ESM.pdf]

SUPPLEMENTARY INFORMATION

Table of contents:

Supplementary Fig. 1: Cryo-EM processing overview.

Supplementary Fig. 2: Resolution and model validation.

Supplementary Fig. 3: SILNAS of human mitoribosomal rRNA.

Supplementary Fig. 4: Complete rRNA sequence showing rRNA modifications determined by SILNAS.

Supplementary Fig. 5: Comparison of polyamine densities with antibiotic-treated cells (PDB ID 6ZM5).

Supplementary Fig. 6: Comparison of protein elements involved in mRNA binding with *E. coli.*

Supplementary Fig. 7: Frequency of Y-N-C-Y motif in the human mitochondrial mRNA transcripts.

Supplementary Fig. 8: Interactions of uS12m with codon in the A-site.

Supplementary Fig. 9: Examples of densities and coordination of ions K^+ and Mg^{2+}

Supplementary Fig. 10: Mitoribosomal proteins involved in mRNA binding.

Supplementary Fig. 11: mRNA-rRNA interactions in the decoding center and roles of modifications.

Supplementary Fig. 12: Analysis of nucleotide binding by mS29 and its role in mitoribosome function.

Supplementary Table 1: Data collection and model statistics.

Supplementary Table 2: Summary of cofactors modeled in human mitoribosome.

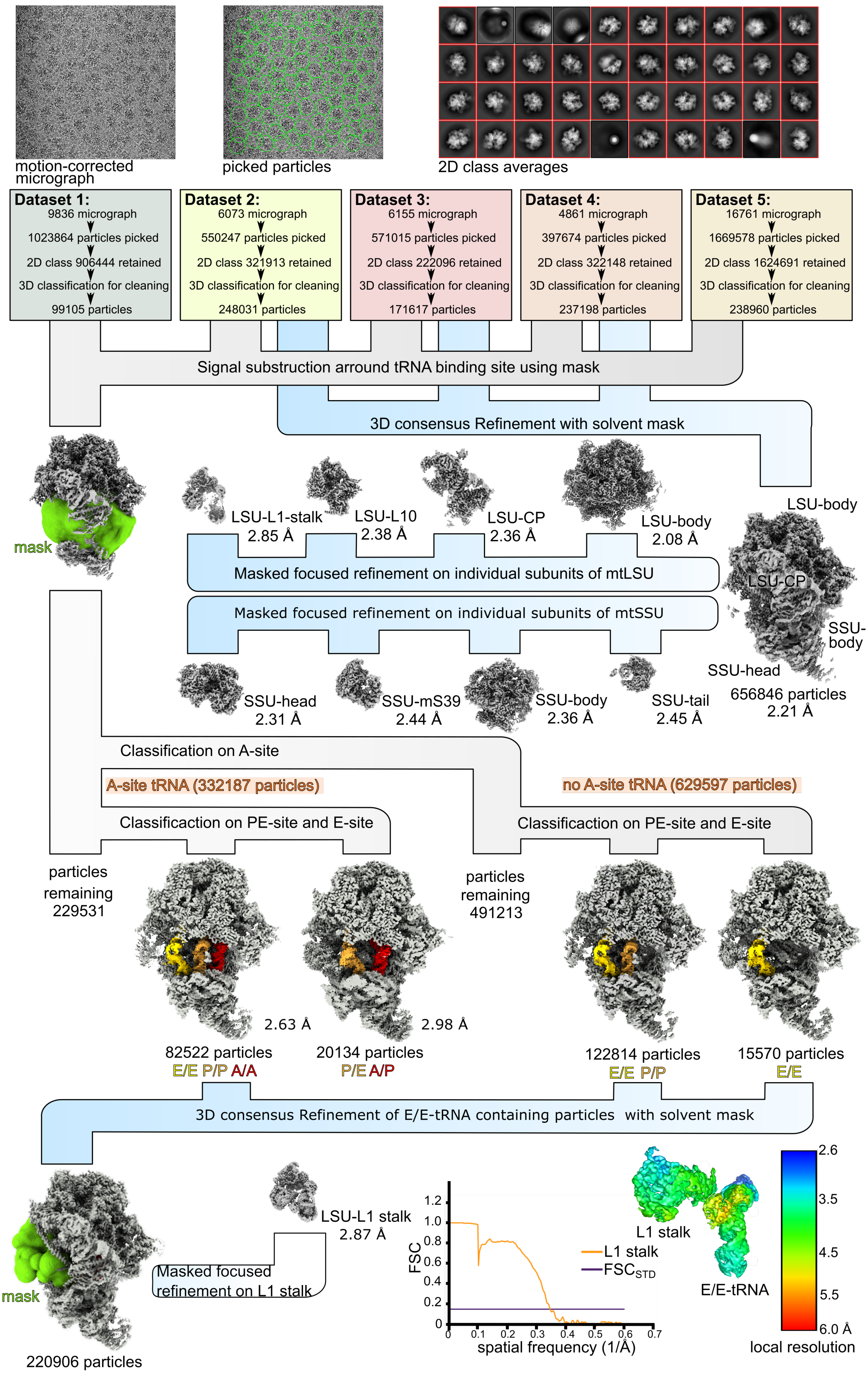

Supplementary Fig. 1: Cryo-EM processing overview.

Data processing scheme for classical (A/A P/P E/E) and hybrid (A/P P/E) states. Bottom panel, local-masked refinements, resolution and model validation for the L1 stalk. A total of 43686 movies were recorded and analyzed, particles that were not mitoribosomes were discarded by classification, since they cannot contribute to reconstruction. Consensus map was produced from 656846 particles. The classical A/A P/P E/E and hybrid A/P P/E maps were obtained from 82522 and 20134 particles, respectively. Cryo-EM structures were successfully obtained from 5 preliminary datasets.

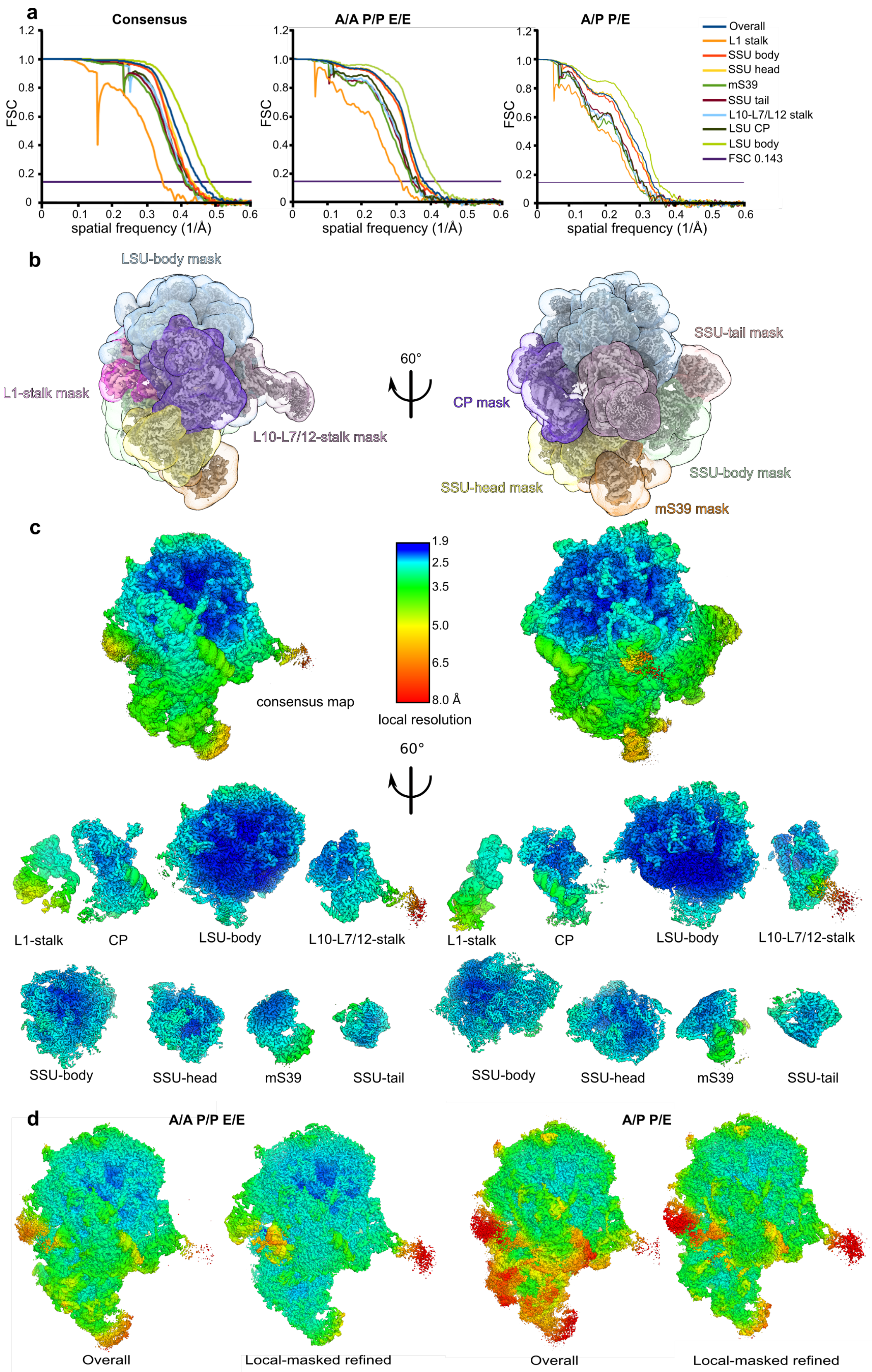

Supplementary Fig. 2: Resolution and model validation.

a, Fourier Shell Correlation curves of the half maps and local-masked refinements. **b,** Binary masks used for local-masked refinements. **c,** Local-masked refinements colored by local resolution. **d,** Overall maps for classical and hybrid states and their corresponding local-masked refined maps fitted and colored by local resolution. Cryo-EM map resolution estimates by Fourier Shell Correlation were performed using half- maps from random half-sets.

a

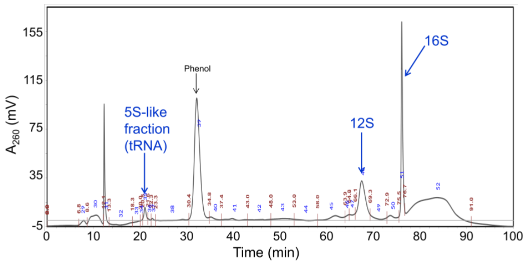

b

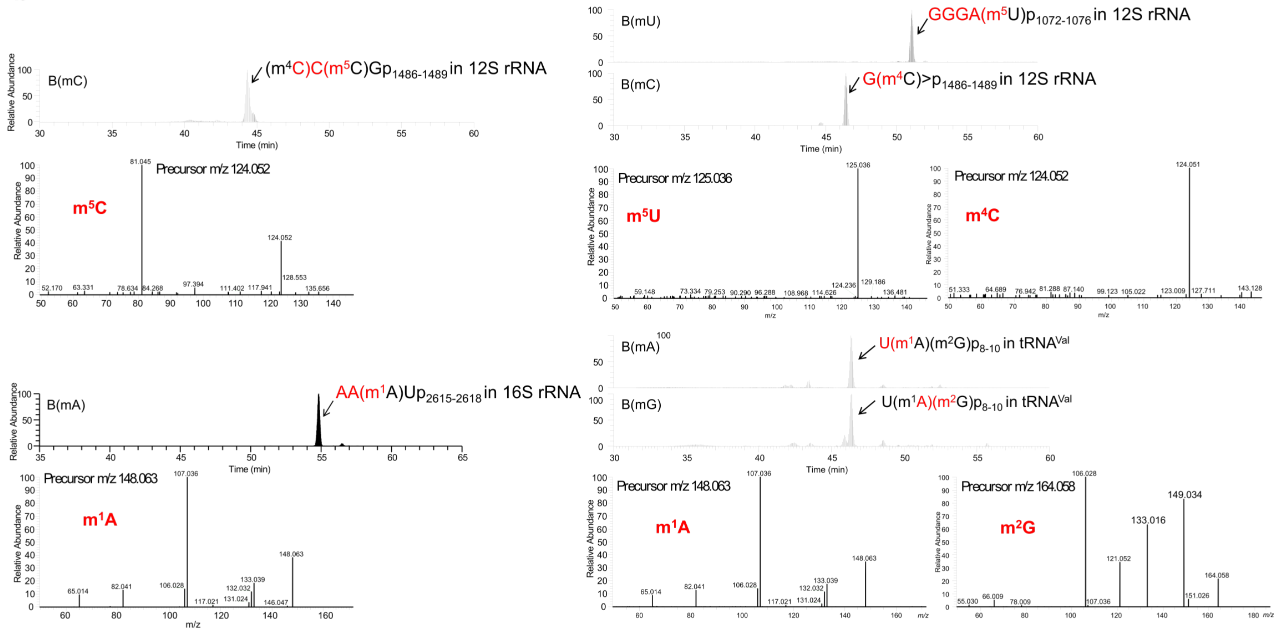

c

Genome # (NC_012920.1)	rRNA name	Position of PTM	rRNA modification name	Occupancy
1610	CP-tRNA^{Val}	9	1-methyladenosine	>78
1611	CP-tRNA^{Val}	10	N2-methylguanosine	>78
1638	CP-tRNA^{Val}	39	pseudouridine	89
838	12S rRNA	191	pseudouridine	17
946	12S rRNA	299	pseudouridine	18
1076	12S rRNA	429	5-methyluridine	97
1207	12S rRNA	560	pseudouridine	37
1257	12S rRNA	610	pseudouridine	29
1270	12S rRNA	623	pseudouridine	7
1486	12S rRNA	839	N4-methylcytidine	96
1488	12S rRNA	841	5-methylcytidine	94
1583	12S rRNA	936	N6,N6-dimethyladenosine	99
1584	12S rRNA	937	N6,N6-dimethyladenosine	99
2617	16S rRNA	947	1-methyladenosine	99
2815	16S rRNA	1145	2´O-methylguanosine	52
3034	16S rRNA	1364	pseudouridine	11
3039	16S rRNA	1369	2´O-methyluridine	96
3040	16S rRNA	1370	2´O-methylguanosine	100
3067	16S rRNA	1397	2´O-methyluridine	98

Supplementary Fig. 3: SILNAS of human mitoribosomal rRNA.

a. Reversed phase-LC isolation of the rRNAs extracted from the HEK 293 mitoribosome (10 μg) applied to a PLRP-S 4000A column (4.6 × 150 mm, 10 μm, Agilent Technologies). **b.** Selective detection and identification of mono-methylated nucleoside isomers in human mitochondrial rRNAs. The HEK293 12S rRNA, 16S rRNA and CP-tRNA^{Val} (each 200 fmol) were digested with RNase T1 or RNase A, and the products subjected to LC-MS with an in-source fragmentation energy of 70 eV. The signals initially extracted for the second step were at m/z 148.06, 164.06, 125.04, and 124.05 (mass tolerance, +/-5 ppm) for B(mA), B(mG), B(mU) and B(mC), respectively. The extracted chromatograms of the oligonucleotides containing the mono-methylated nucleobase dissociated from RNase A and RNAse T1 digestion of the mitochondrial 12S rRNA (top left and top right panels) and 16S rRNA (bottom left and bottom right panels) are shown, respectively. Pseudo-MS3 spectra of the mono-methylated nucleobases are shown below each. The sequences assigned by Ariadne are shown next to their signals (arrow). The assigned mono-methylated isomers are shown in red. The spectrum was obtained from the m^2G anion of the oligonucleotide because the ratio of the signal heights at m/z 133.016 and 149.034 are 0.75. **c.** Table lists all the HEK 293 mt-rRNA modifications identified with their respective occupancies.

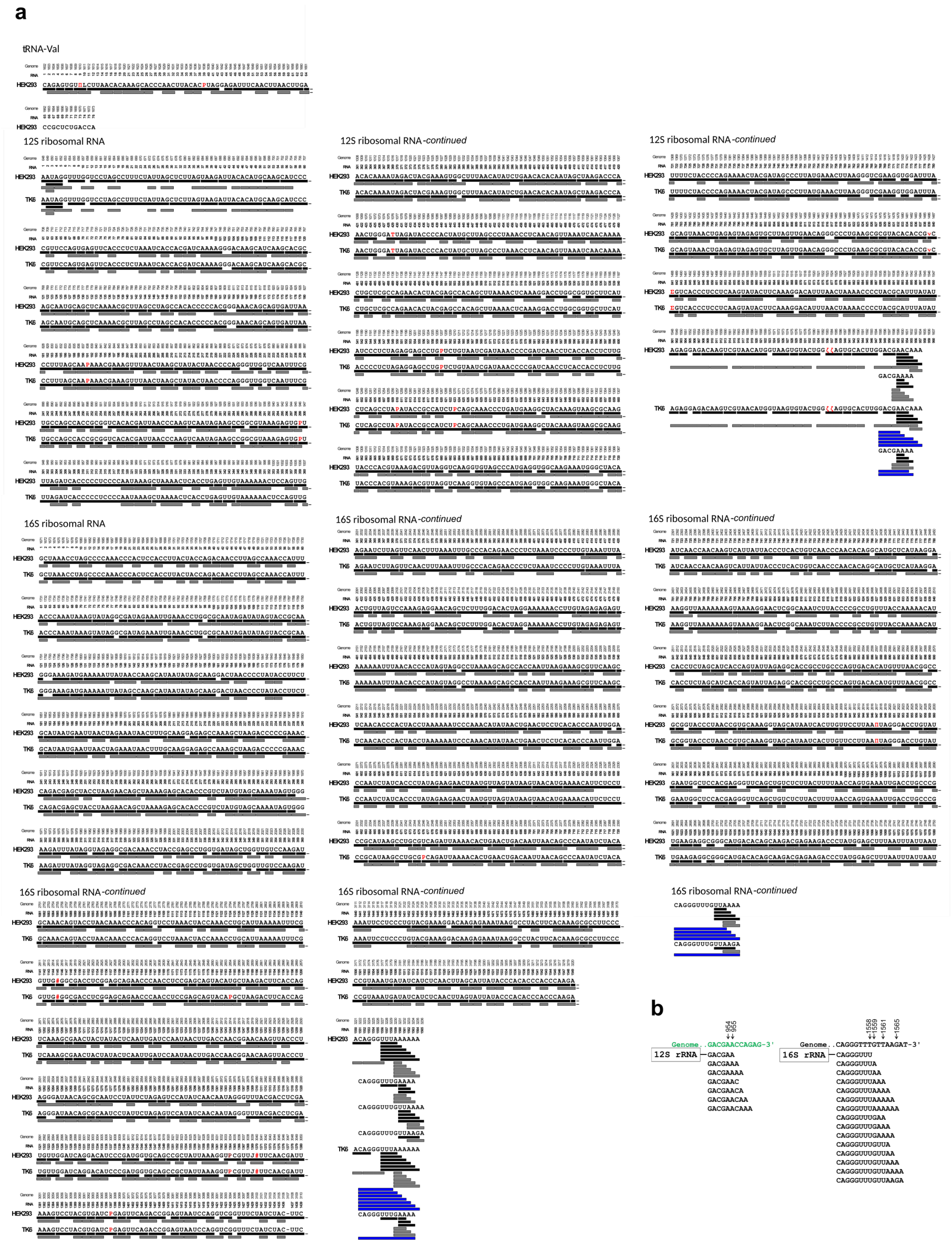

Supplementary Fig. 4: Complete rRNA sequence showing rRNA modifications determined by SILNAS.

a, The complete sequence (numbered according to NC_012920.1) and modifications of the human mitochondrial rRNAs and the fragments used for the structural analysis. Digestion fragments produced by RNase T1 (Black solid); RNase A (black shaded bars) and RNase H (Blue solid bars) are indicated. All fragments were identified by Ariadne software (see Methods). Modified

nucleotides (red letters) and their abbreviations are summarized as follows: P, pseudouridine; Π, 1-methyladenosine; ζ, N6,N6-dimethyladenosine; ν, N4-methylcytidine; Σ, 5-methylcytidine; #, 2′-O-methylguanosine; L, N2-methylguanosine; J, 2′-O-methyluridine; T, 5-methyluridine. **b,** 3' end of 12S and 16S mt-rRNAs show hetero sequences. Mitochondrial genome sequence (NC_012920.1) around 3' end of 12S and 16S are indicated as green text. The transcription termination points are indicated with the residue number and arrow. The hetero 3' end of 12S and 16S mitochondrial rRNAs detected in this study are shown in plain text.

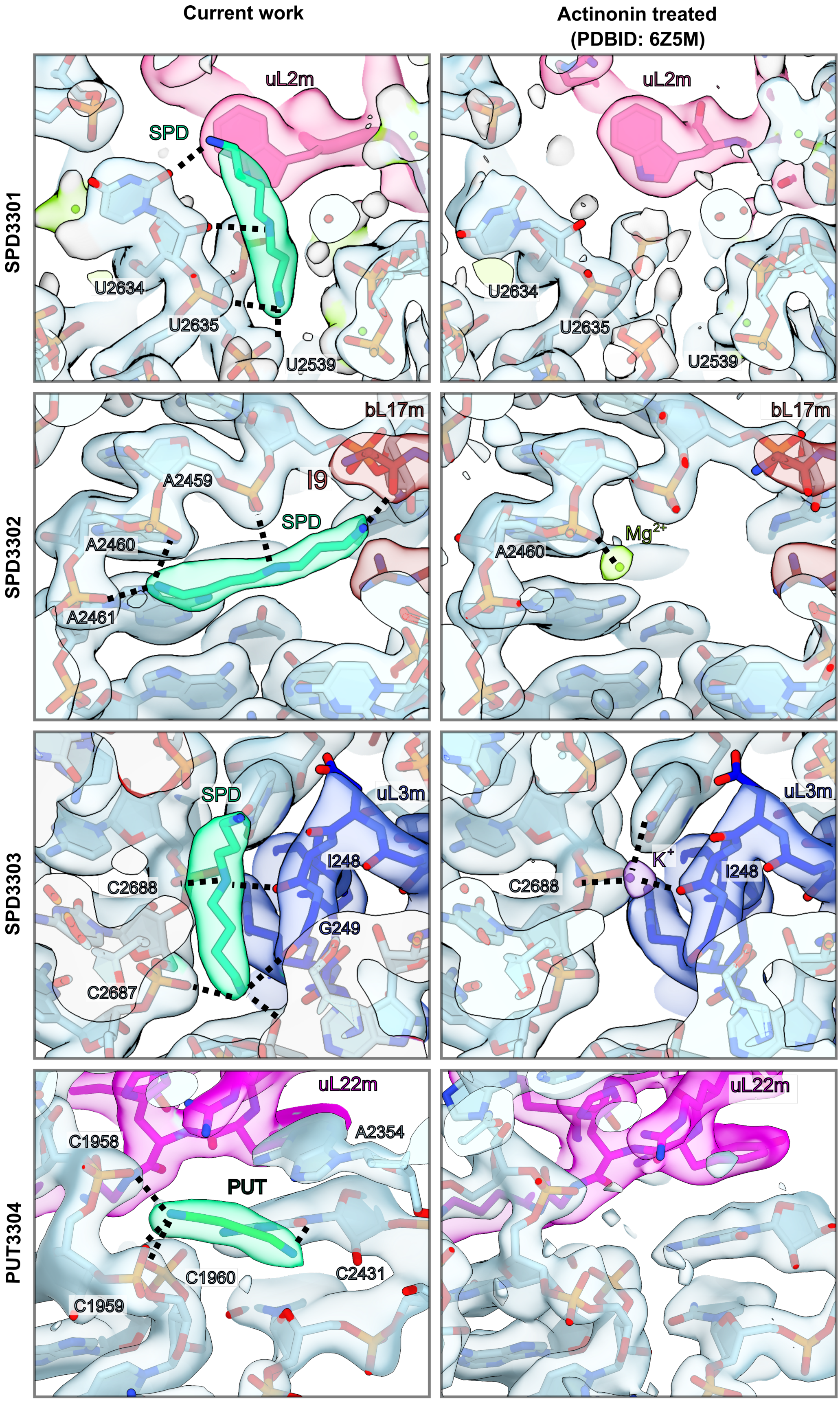

Supplementary Fig. 5: Comparison of polyamine densities with antibiotic-treated cells (PDB ID 6ZM5). Densities (colored by chain) corresponding to spermidine and putrescine are absent upon antibiotic treatment [71]. Absence of putrescine is further associated with a conformational rearrangement of the residue A2354. Interactions are shown with dashed lines.

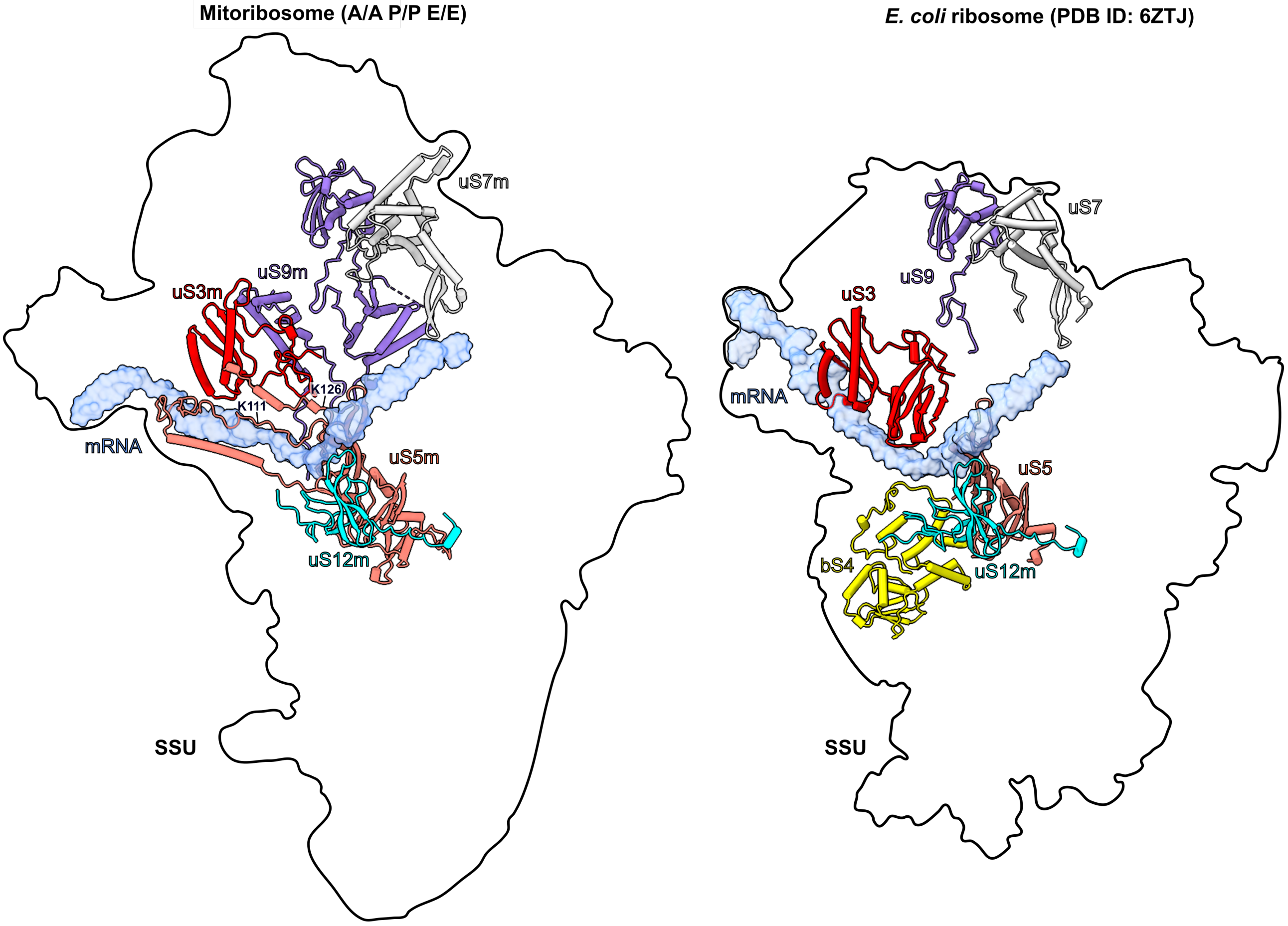

Supplementary Fig. 6: Comparison of protein elements involved in mRNA binding with *E. coli*.

Proteins uS5m, uS7m, uS9m and uS12m (cartoon), mRNA (light-blue surface) from the mitoribosome (left) compared with their bacterial counterparts from *E. coli* [66] (PDB ID 6ZTJ; right). The residues K111 and K126 mark the poly-basic stretch of uS5m. Mitochondria-specific extensions of uS5m and uS9m are positioned along the mRNA channel.

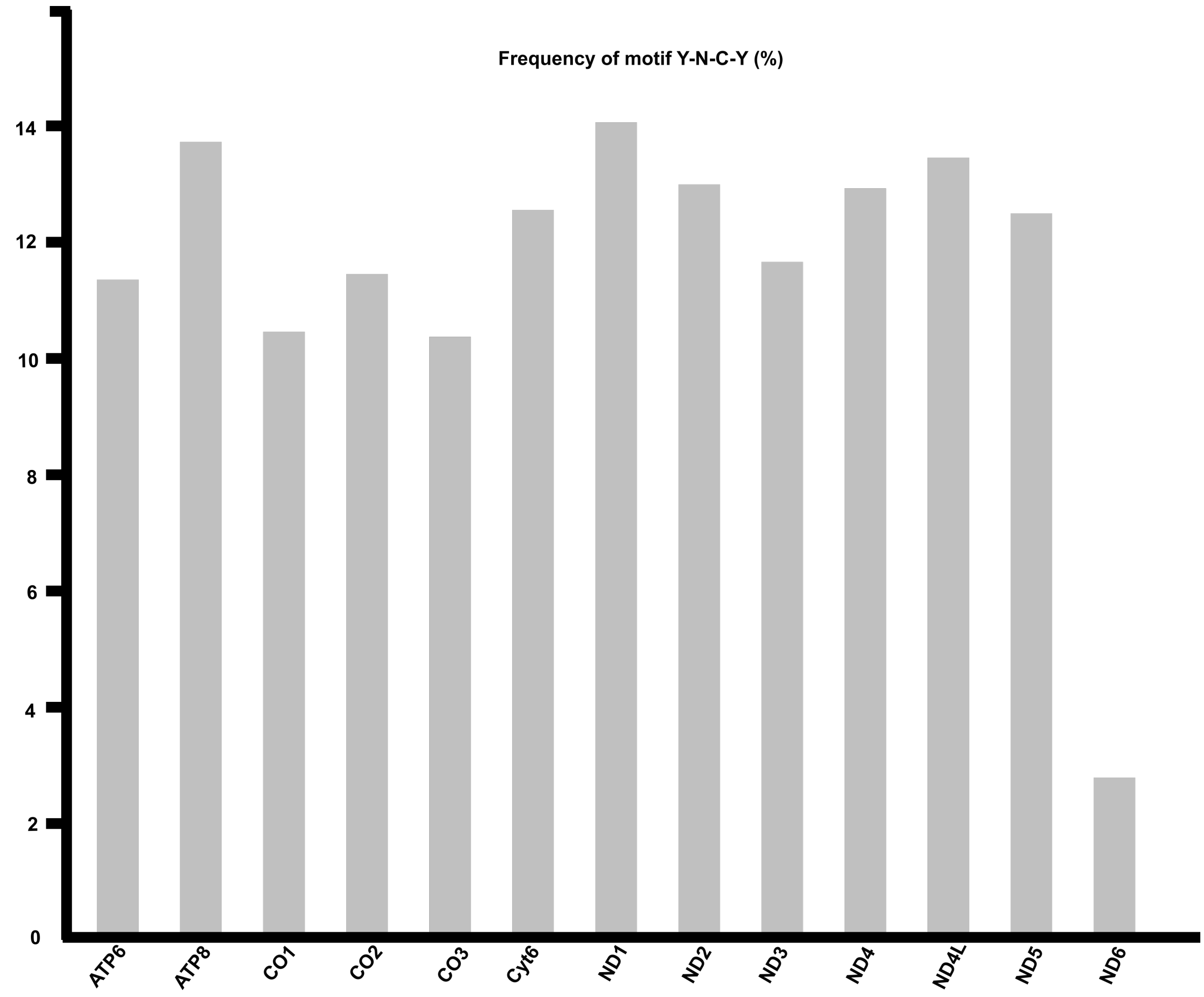

Supplementary Fig. 7: Frequency of Y-N-C-Y motif in the human mitochondrial mRNA transcripts.

Percentage frequency of Y-N-C-Y motif in each of the 13 mitochondrial mRNAs (light grey bars).

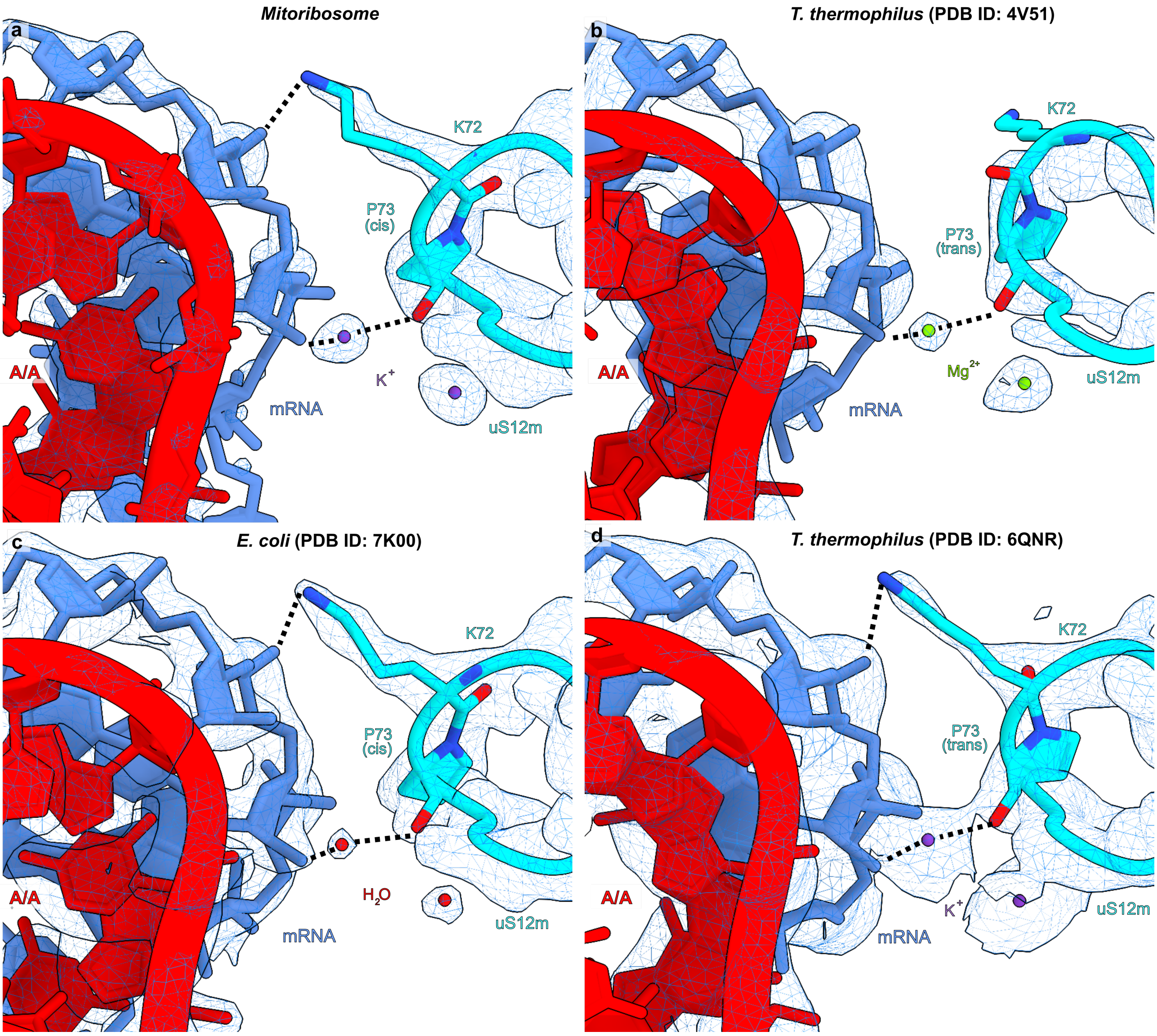

Supplementary Fig. 8: Interactions of uS12m with codon in the A-site.
a, In the mitoribosome at 2.2 Å resolution, P73 is in *cis*, and it coordinates a K^+ ion. K72 side-chain interacts with U9 of the A-site codon. **b,** In *T. thermophilus* ribosome (PDB ID 4V51) at 2.8 Å resolution [52], the densities are assigned as Mg^{2+}, P73 is in *trans*, and K72 is pointing away from the A-site codon. **c,** In *E. coli* ribosome (PDB ID 7K00) at 2.0 Å resolution [48], two water molecules have been modeled, and P73 is in *cis*. **d,** In *T. thermophilus* ribosome (PDB ID 6QNR) at 3.1 Å resolution [53], the densities were assigned as K^+ based on long wavelength X-ray diffraction.

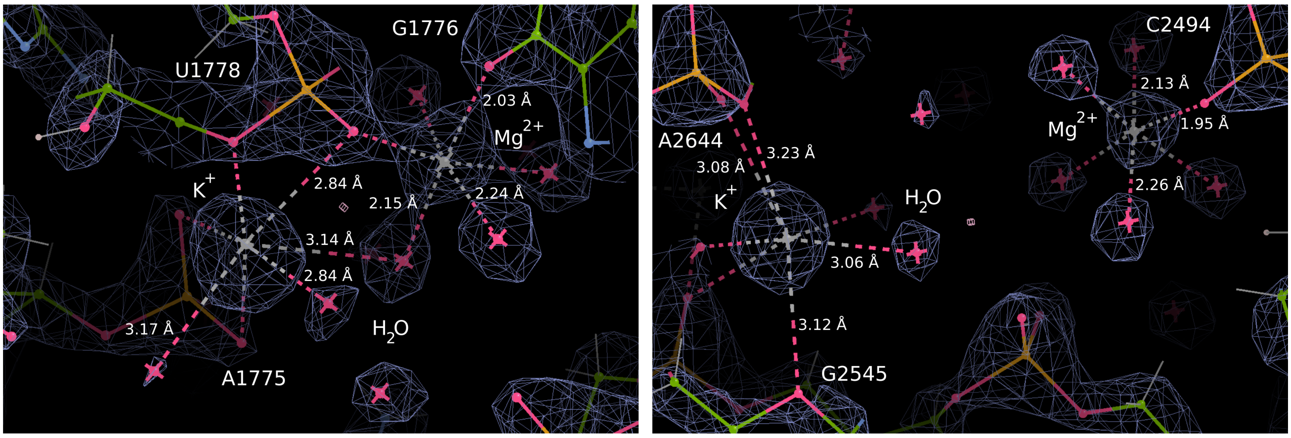

Supplementary Fig. 9: Examples of densities and coordination of ions K^+ and Mg^{2+}. Ions and water molecules are shown with their densities. While Mg^{2+} ions have a consistent octahedral coordination with coordinating atoms located around 2 Å away, the corresponding distances for K^+ are closer to 3 Å. In total there are 54 K^+ and 206 Mg^{2+} ions modeled in the structure of the human mitoribosome (PDB 7QI4).

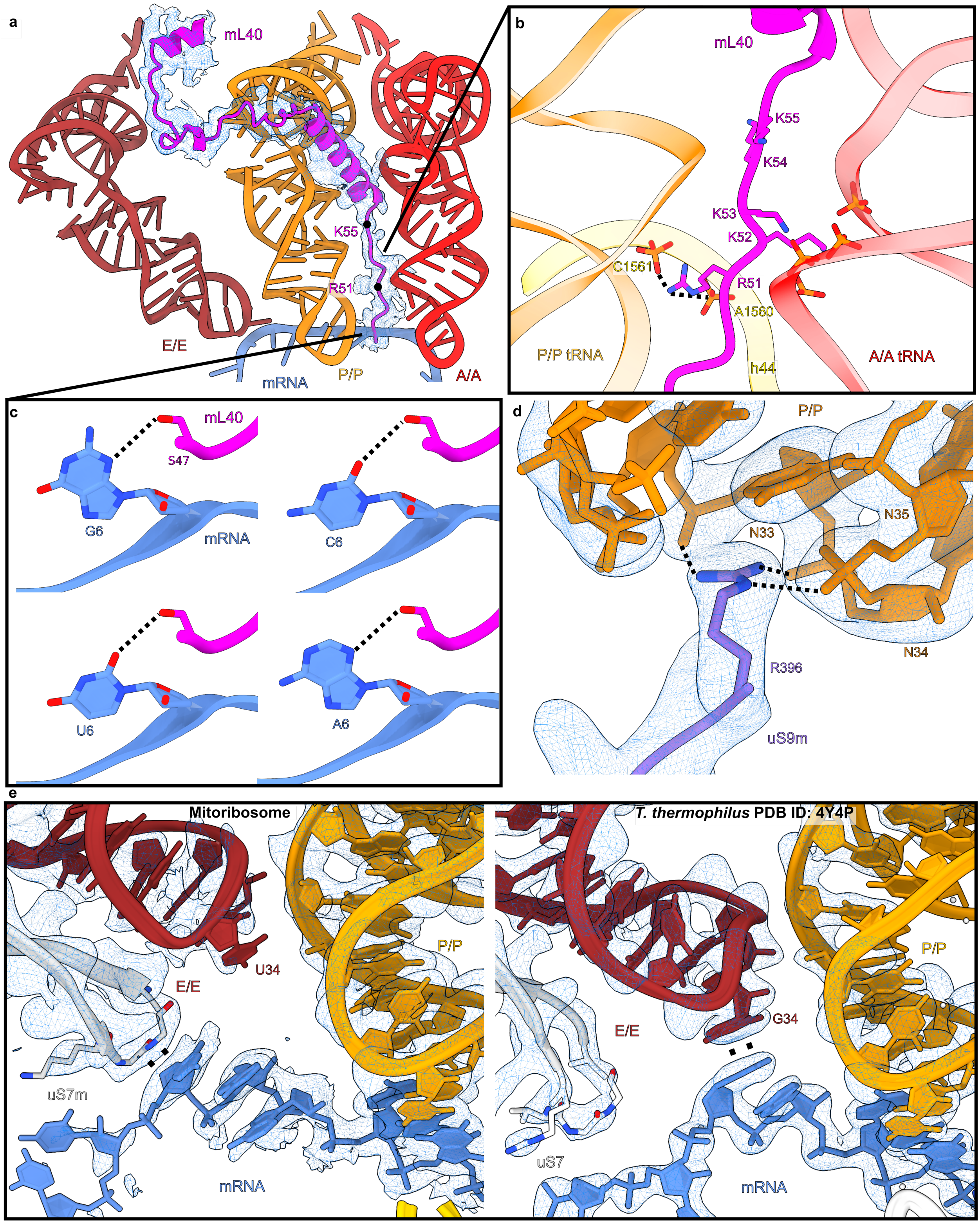

Supplementary Fig. 10: Mitoribosomal proteins involved in mRNA binding.
a, N-terminal region of mL40 lodged between A- and P-tRNA. **b,** basic residues 51-55 of mL40 interact with tRNA phosphate backbone. R51 forms salt bridges with A1560 and C1561 phosphates in h44. **c,** The mRNA residue at position 6 is mutated in Coot v0.9 to depict potential H-bond between S47 side chain and base regardless of base identity. **d,** uS9m C-terminal residue R396 interact with P/P-tRNA anticodon stem-loop. **e,** E-tRNA does not directly interact with mRNA in the mitoribosome. Instead, Gly164 and Gly165 of uS7m stack against mRNA nucleotide base. This is in contrast to the bacterial ribosome [62] (PDB ID 4Y4P) where E-tRNA nucleotide at position 34 stacks with mRNA nucleotide base.

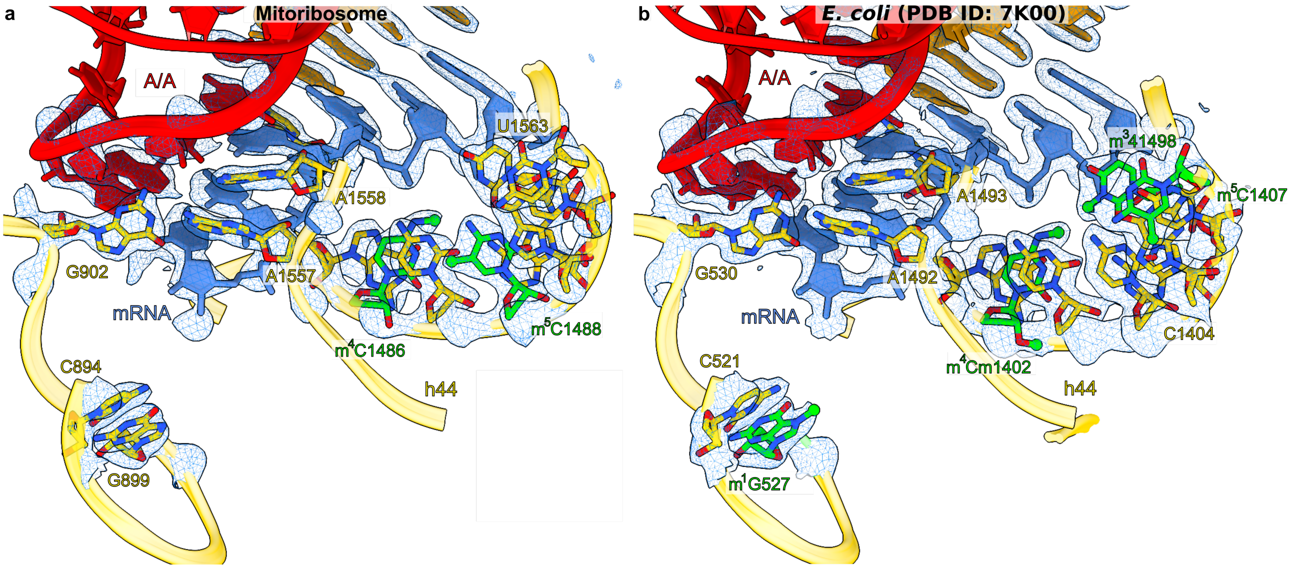

Supplementary Fig. 11: mRNA-rRNA interactions in the decoding center and roles of modifications.

a, Mitoribosome in the classical state: rRNA yellow, mRNA blue, tRNA red, modified nucleotides are labeled and colored lime, cryo-EM density in blue mesh. **b,** *E. coli* ribosome [48] (PDB ID 7K00), the same color scheme as in (a). Differences are observed along the mRNA codons: m^{4}C1486 (methylated m^{4}Cm1402 in *E. coli*); m^{5}C1488 (unmodified C1404 in *E. coli*), U1563 (N3-methylated m^{3}U1498 in *E. coli*). Conserved nucleotides at the decoding center G902, A1557, A1558 interact directly with A-site tRNA anti-codon, equivalent to the corresponding residues G530, A1492 and A1493 in *E. coli*.

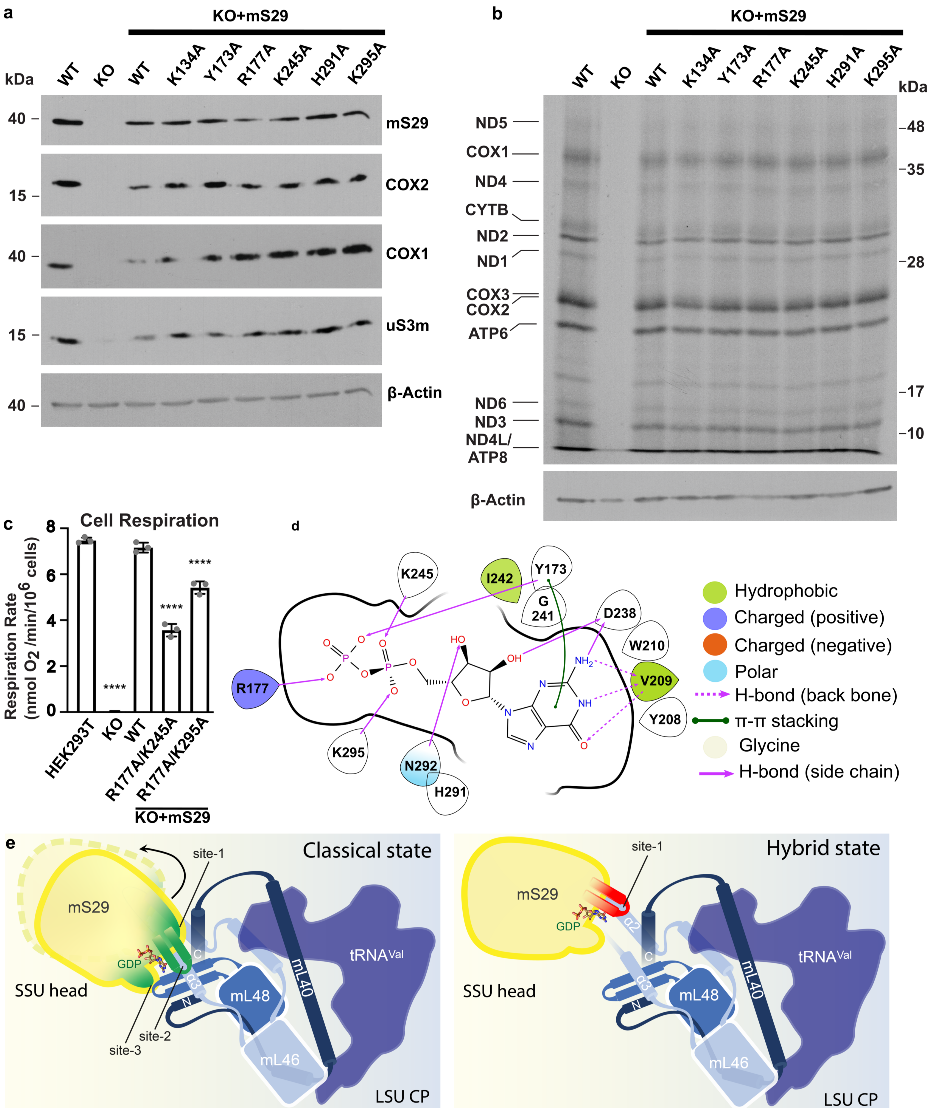

Supplementary Fig. 12: Analysis of nucleotide binding by mS29 and its role in mitoribosome function. a, Immunoblot analysis comparing steady-state levels of proteins using whole cell protein lysates from HEK293T (WT), *mS29*-KO (KO), and *mS29*-KO cells stably expressing either WT mS29 or single mutants in the ATP binding site (K134A) or GDP binding site (Y173A, R177A, K245A, H291A, K295A) under an attenuated CMV6 promoter (Δ5pCMV6). COX1 and COX2 were used as surrogates of mitoribosome function, uS3m as a control for the SSU, and β-ACTIN as a loading control. Single mutations of mS29 did not have an effect on the steady-state levels of mitochondrial proteins. **b,** Metabolic labeling of mitochondrially translated peptides in whole cells from the indicated cell lines using ^{35}S-methionine for 15 min. Immunoblotting for β-ACTIN was used as a loading control. Newly synthesized peptides are identified on the left. Experiments a,b were carried out once **c,** Cyanide-sensitive endogenous cell respiration rate measured polarographically using a Clark electrode in the indicated cell lines. Cell respiration rates are presented as nmol of O_2 consumed per minute per one million cells. One-way analysis of variance (ANOVA) was performed, comparing the mean of each cell line with the mean of the mS29-KO cell line reconstituted with WT mS29 or mutants, followed by Holm-Sidak's multiple comparisons test (two-tailed). Data represents the mean ± SD of three independent repetitions (n=3; *p<0.05, **p<0.01; ***p<0.001, ****p<0.0001). The adjusted p-value for HEK293T vs. KO, vs. KO + mS29 (R177A/K245A), and vs. KO + mS29 (R177A/K295A) was < 0.0001, and vs. KO + mS29 (WT) = 0.1187. **d,** 2D diagram of GDP interactions. **e,** Schematic depiction of intersubunit contacts mediated by mS29.

	Monosome (consensus) PDB: 7QI4 EMD-13980	Monosome (A/A P/P E/E) PDB: 7QI5 EMD-13981	Monosome (A/P P/E) PDB: 7QI6 EMD-13982
**Data collection and processing**			
Electron microscope	Titan Krios	Titan Krios	Titan Krios
Camera	K2 Summit (counting mode)	K2 Summit (counting mode)	K2 Summit (counting mode)
Magnification	165,000	165,000	165,000
Voltage (kV)	300	300	300
Electron exposure ($e^{-}/Å^2$)	29–32	29–32	29–32
No. of frames	20	20	20
Defocus range (μm)	−0.6 to −2.8	−0.6 to −2.8	−0.6 to −2.8
Pixel size (Å)	0.83	0.83	0.83
Symmetry imposed	C_1	C_1	C_1
Final particle number (no.)	656,846	82,522	20,134
Map resolution (Å) (Overall/ LSU-body/ CP/ L10-L12-stalk/ L1-stalk/ SSU-body/ SSU-head/ mS39/ SSU-tail/ mS39-LRPPRC-SLIRP)	2.21/ 2.08/ 2.36/ 2.38/2.89/ 2.31/ 2.36/ 2.45/ 2.44/ –	2.63/ 2.44/ 2.78/ 2.84/3.20/ 2.75/2.72/ 2.93/ 2.91/ –	2.98/ 2.84/ 3.25/3.29/3.53/3.07/3.03/3.47/3.39/ –
FSC threshold	0.143	0.143	0.143
Map resolution range (Å)	1.9-8.0	2.1-8.0	2.4-8.0
**Refinement**			
Initial model used (PDB code)	6ZSG, 6RW4	6ZSG, 6RW4	6ZSG, 6RW4
Model resolution (Å)	2.10	2.50	2.80
Model to map CC (CC_{volume})	0.84	0.84	0.81
FSC threshold	0.5	0.5	0.5
Map-sharpening *B* factor ($Å^2$) (Overall/ LSU-body/ CP/ L10-L12-stalk/ L1-stalk/ SSU-body/ SSU-head/ mS39/	-38/ -34/ -53/-55/-74/ -43/ -45/ -52/ -52/ –	-36/ -28/ -52/-53/-60/ -39/ -42/ -51/ -51/ –	-42/ -21/ -50/-59/-40/ -35/ -49/ -64/ -57/ –
Model composition			
Non-hydrogen atoms	187689	189567	183520
Hydrogen atoms	152459	154149	153182
Protein chains	88	88	88
RNA chains	7	7	6
Protein residues (non-modified/ *N*-acetylAla/ *N*-acetylSer/ *N*-acetylThr O^1-methylisoAsp)	14790/3/1/1/1	14891/3/1/1/1	14879/3/1/1/1
RNA residues (non-modified/ mG/ mU/ m^1A/ m^2G/ψ /m^4C/ m^5C/ m^5U/ $m^6{}_2A$)	2750/2/ 1/ 2/ 1/ 2/ 1/ 1/ 1/ 2	2814/2/ 1/ 2/ 1/ 2/ 1/ 1/ 1/ 2	2744/ 2/1/ 2/ 1/ 2/ 1/ 1/ 1/ 2
Ligands (ATP/ GDP/ NAD/ 2Fe-2S/ spermine/ spermidine/ putrescine)	1/ 1/ 1/ 3 /1 /4/ 1	1/ 1/ 1/ 3 /1 /4/ 1/	1/ 1/ 1/ 3 /1 /4/ 1
Ions (Zn^{2+}/ K^+/ Mg^{2+})	3/ 54/ 206	3/ 54/ 208	3/ 54/ 202
Waters	8,001	7,714	3,304
Mean atomic *B*-factor ($Å^2$)			
Protein	42.146	49.50	33.04
RNA	41.81	45.12	32.94
Ligand	27.34	26.81	16.35
Water	25.96	24.50	11.57
**Validation**			
Ramanchandran plot (%)			
Outliers	0.03	0.03	0.02
Allowed	1.45	1.50	1.54
Favored	98.52	98.47	98.44
Clash score	1.48	2.20	2.03
RMSD			
Bonds (Å)	0.002	0.004	0.002
Angles (°)	0.4531	0.457	0.434
Rotamer outliers (%)	0.00	0.00	0.00
C_β outliers (%)	0.00	0.00	0.00
CaBLAM outliers (%)	0.85	0.77	0.81

Supplementary Table 1: Data collection and model statistics.

Cofactors	Protein/rRNA (Interacting Residues)	Local resolution (Å)	Notes
mt-SSU			
ATP	mS29 (M100, G131, G133, K134, T135, L136, S311, T313)	2.28	Previously, reported as GDP [2]. Strong density for γ-phosphate in both monosome and SSU [45] structures indicates a lack of hydrolysis activity. Solvent inaccessible.
GDP	mS29 (Y173, R177, V209, D238, I242, K245, H291, N292, K295)	2.65	Stabilizes inter-subunit contacts between mS29 of SSU and mL40 and mL46 of LSU in classical and hybrid states. Solvent accessible.
NAD	12S rRNA (A781, A782, U948, A1046, C1048)	2.17	Binds together with spermine at rRNA h20 to stabilize rRNA insertion of C1048 [45].
	uS15m (Y196)		
2Fe-2S	uS18m (C65, C68, C100)	2.17	Stabilized mito-specific extensions compensate for the loss of rRNA h26 [45] to bridge rRNA h20-22 and h25. Potentially, stabilizes h24 that contributes to P- and E-sites
	bS6m (C105)		
2Fe-2S	mS25 (C139, C141, C149)	2.33	Stabilized mS25 compensates for the loss of rRNA h21 [45] to bridge the gap between rRNA helices h4 and h20.
	bS16m (C26)		
Spermine	12S rRNA (U944, G945, U946, A1110, U1044, G1045, A1047)	2.16	Binds together with NAD at rRNA h20 and interacts with back-bone phosphate of C1048 [45].
mt-LSU			
2Fe-2S	mL66 (C70, C73, C108)	2.22	Bridges mL66 and mito-specific extension of uL10m potentially stabilizing the L12-stalk. Previously modeled as Zn^{2+} [2].
	uL10m (C64)		
Spermidine 3301	16S (C2538, A2539, U2634, G2635)	2.02	Absent in monosome from actinonin treated HEK cells (PDB ID 6ZM5) [71]
	uL2m (W275)		
Spermidine 3302	16S (A2458, A2459, A2460, A2461)	1.97	Replaced by Mg^{2+} in monosome from actinonin treated HEK cells [71]
	bL17m (I9)		

Spermidine 3303	16S (C2687, C2688, A2696, U3104)	1.93	Replaced by K^+ in monosome from actinonin treated HEK cells [71]
	uL3m (I248, G249)		
Putrescine 3304	16S rRNA (G1958, A1960, C2431)	1.94	Absent in monosome from actinonin treated HEK cells [71]

Supplementary Table 2: Summary of cofactors modeled in human mitoribosome.

Original blot image

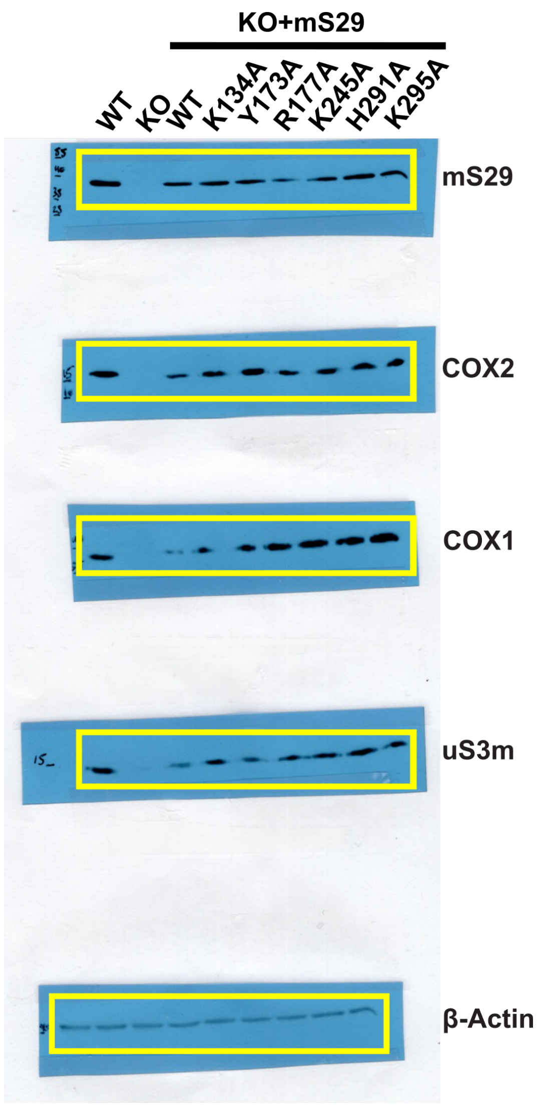

The representative blot is shown in Supplementary Fig. 12a

Original blot image

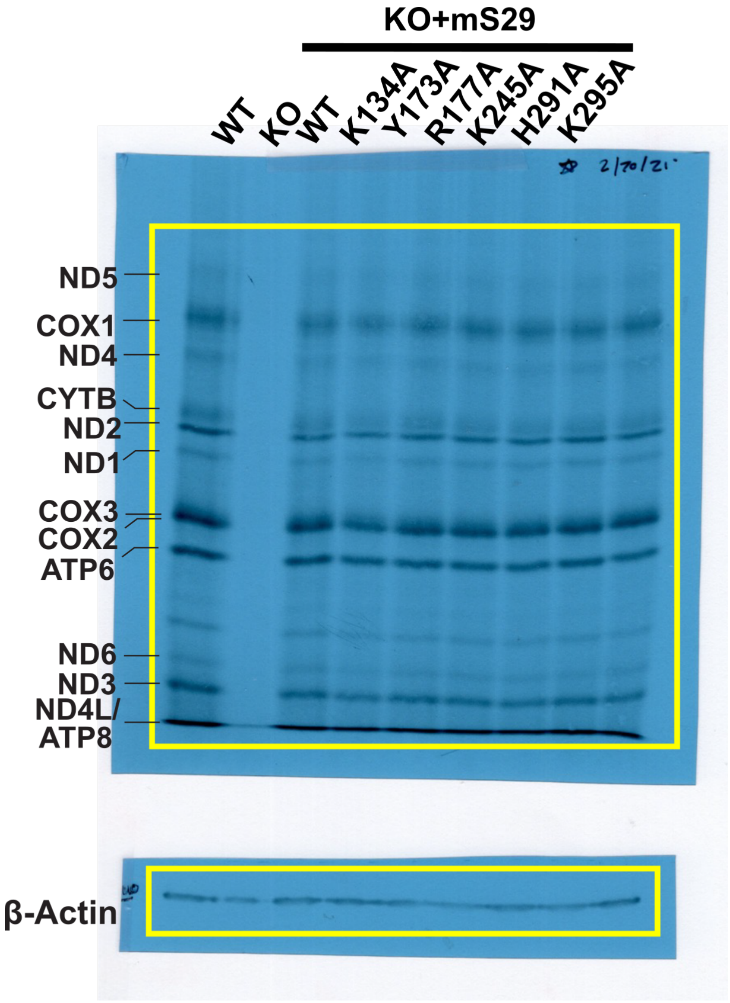

The representative blot is shown in Supplementary Fig. 12b
