# Supplementary material for: Mitoribosome structure with cofactors and modifications reveals mechanism of ligand binding and interactions with L1 stalk
Source: Nat Commun. 2024 May 20;15:4272. doi: 10.1038/s41467-024-48163-x (PMC11106087; doi:10.1038/s41467-024-48163-x)
Supplement: Supplementary file 3 — Description of Additional Supplementary Files [file 41467_2024_48163_MOESM3_ESM.pdf]

File name: Supplementary Movie 1

Description: The mRNA path and mitoribosome-specific components involved in mRNA recognition.

File name: Supplementary Movie 2

Description: Simulation of P/E hybrid-state formation in the human mitoribosome.
