# Supplementary material for: Mitoribosome structure with cofactors and modifications reveals mechanism of ligand binding and interactions with L1 stalk
Source: Nat Commun. 2024 May 20;15:4272. doi: 10.1038/s41467-024-48163-x (PMC11106087; doi:10.1038/s41467-024-48163-x)
Supplement: Supplementary file 6 — Reporting Summary [file 41467_2024_48163_MOESM6_ESM.pdf]

Corresponding author(s): Alexey Amunts

Last updated by author(s): Mar 24, 2024

Reporting Summary

Nature Portfolio wishes to improve the reproducibility of the work that we publish. This form provides structure for consistency and transparency in reporting. For further information on Nature Portfolio policies, see our Editorial Policies and the Editorial Policy Checklist.

Statistics

For all statistical analyses, confirm that the following items are present in the figure legend, table legend, main text, or Methods section.

n/a	Confirmed	
☐	☒	The exact sample size (*n*) for each experimental group/condition, given as a discrete number and unit of measurement
☐	☒	A statement on whether measurements were taken from distinct samples or whether the same sample was measured repeatedly
☐	☒	The statistical test(s) used AND whether they are one- or two-sided<br>*Only common tests should be described solely by name; describe more complex techniques in the Methods section.*
☒	☐	A description of all covariates tested
☒	☐	A description of any assumptions or corrections, such as tests of normality and adjustment for multiple comparisons
☐	☒	A full description of the statistical parameters including central tendency (e.g. means) or other basic estimates (e.g. regression coefficient) AND variation (e.g. standard deviation) or associated estimates of uncertainty (e.g. confidence intervals)
☐	☒	For null hypothesis testing, the test statistic (e.g. *F*, *t*, *r*) with confidence intervals, effect sizes, degrees of freedom and *P* value noted<br>*Give P values as exact values whenever suitable.*
☒	☐	For Bayesian analysis, information on the choice of priors and Markov chain Monte Carlo settings
☒	☐	For hierarchical and complex designs, identification of the appropriate level for tests and full reporting of outcomes
☒	☐	Estimates of effect sizes (e.g. Cohen's *d*, Pearson's *r*), indicating how they were calculated

Our web collection on statistics for biologists contains articles on many of the points above.

Software and code

Policy information about availability of computer code

Data collection	The datasets were collected EPU 1.9 software on FEI Titan Krios (FEI/Thermofischer) transmission electron microscope operated at 300 keV with a slit width of 20 eV on a GIF quantum energy filter (Gatan). A K2 Summit detector (Gatan) was used at a pixel size of 0.83 A (magnification of 165,000x) with an exposure rate of 4.26 electrons/pixel/second fractionated over 20 frames. A defocus range of -0.6 to -2.8 µm was used.
Data analysis	Movie frames were aligned and averaged by global and local motion corrections by the program RELION3.0. Contrast transfer function (CTF) parameters were estimated by gctf. Particles were picked and classified by RELION. The models were manually built with Coot 0.9 and stereochemical refinement was performed using phenix.real_space_refine in the PHENIX 1.19 suite. Figures made with ChimeraX. Simulation data analysis is achieved with GROMACSS. 5.1.4 tools. Statistical anlyses were done use GraphPad Prism v8, with either one-way ANOVA or paired Welch's t-tests.

For manuscripts utilizing custom algorithms or software that are central to the research but not yet described in published literature, software must be made available to editors and reviewers. We strongly encourage code deposition in a community repository (e.g. GitHub). See the Nature Portfolio guidelines for submitting code & software for further information.

Data

Policy information about availability of data

All manuscripts must include a data availability statement. This statement should provide the following information, where applicable:

- Accession codes, unique identifiers, or web links for publicly available datasets
- A description of any restrictions on data availability
- For clinical datasets or third party data, please ensure that the statement adheres to our policy

The atomic coordinates were deposited in the RCSB Protein Data Bank, and EM maps have been deposited in the Electron Microscopy Data bank under accession numbers: 7QI4 and EMD- 13980 (consensus monosome), 7QI5 and EMD-13981(classical state), 7QI6 and EMD-13982 (hybrid state).
The atomic coordinates that were used in this study: 3U4M (uL1 from T. thermophilus), 4V51 (T. thermophilus ribosome with mRNA, tRNA), 4V9D (E. coli ribosome), 4Y4P (T. thermophilus ribosome with A-, P-, E-site tRNAs), 6QNR (T. thermophilus ribosome with experimentally assigned K+ ions) 6RW4 (mtSSU with mtIF3), 6ZM5 (mitoribosome from actinonin-treated cells) 6ZSG (mitoribosome with mRNA, tRNA), 6ZTJ (E.coli expressome), 6ZTN (E.coli expressome), 7K00 (E. coli ribosome at 2 Å resolution).

Human research participants

Policy information about studies involving human research participants and Sex and Gender in Research.

Reporting on sex and gender	N/A
Population characteristics	*Describe the covariate-relevant population characteristics of the human research participants (e.g. age, genotypic information, past and current diagnosis and treatment categories). If you filled out the behavioural & social sciences study design questions and have nothing to add here, write "See above."*
Recruitment	*Describe how participants were recruited. Outline any potential self-selection bias or other biases that may be present and how these are likely to impact results.*
Ethics oversight	*Identify the organization(s) that approved the study protocol.*

Note that full information on the approval of the study protocol must also be provided in the manuscript.

Field-specific reporting

Please select the one below that is the best fit for your research. If you are not sure, read the appropriate sections before making your selection.

☒ Life sciences ☐ Behavioural & social sciences ☐ Ecological, evolutionary & environmental sciences

For a reference copy of the document with all sections, see nature.com/documents/nr-reporting-summary-flat.pdf

Life sciences study design

All studies must disclose on these points even when the disclosure is negative.

Sample size	A total of 43,786 movies were recorded and analyzed. No statistical analyses has been performed. The number of cryo-EM particles in the single dataset collected was the number of particles available. No predetermined sample size was used for other experiments.
Data exclusions	For cryo-EM structure determination, particles that were not mitoribosomes were discarded by classification, since they cannot contribute to reconstruction.
Replication	Cryo-EM structures were successfully obtained from preliminary datasets. In MD simulations multiple simulation replicas were performed. Overall, consistent results were obtained from different simulation replicas.<br>For biochemical analysis, three repetitions were done using independent biological samples with repeatable results for all main figures. All results were successfully replicated in each experiment.
Randomization	MD simulations did not include randomization. Cryo-EM map resolution estimates by Fourier Shell Correclation were performed using half-maps from random half-sets.
Blinding	MD simulations did not include blinding. N/A to cryo-EM study; raw micrographs or particle images are not categorical data. Particles are randomly assigned into half-sets for image processing; hence no blinding is applicable. All immunoblots, cell respiration measurements, and quantification of data were done by a single researcher (SD). For most instances, blinding was not feasible.

Reporting for specific materials, systems and methods

We require information from authors about some types of materials, experimental systems and methods used in many studies. Here, indicate whether each material, system or method listed is relevant to your study. If you are not sure if a list item applies to your research, read the appropriate section before selecting a response.

Materials & experimental systems

n/a	Involved in the study	
☐	☒	Antibodies
☐	☒	Eukaryotic cell lines
☒	☐	Palaeontology and archaeology
☒	☐	Animals and other organisms
☒	☐	Clinical data
☒	☐	Dual use research of concern

Methods

n/a	Involved in the study	
☒	☐	ChIP-seq
☒	☐	Flow cytometry
☒	☐	MRI-based neuroimaging

Antibodies

Antibodies used

Primary antibodies against the following human proteins were used at the dilutions described here: Beta-Actin (1:2,000 dilution; Proteintech, 66009-1-lg), COXl (1:1,000 dilution; Abeam, ab14705}, COX2 (1:1,000 dilution; Abeam, ab110258), COXSB (1:1,000 dilution; Santa Cruz Biotechnology Inc., sc-374417), mS29/DAP3 (1:1,000 dilution; Sigma-Aldrich, HPA023687)
Horseradish peroxidase-conjugated anti-mouse or anti-rabbit lgG's were used a secondary antibodies (1:10,000 dilution; Rockland lmmunochemicals, 610-103-121 & 611-1302)

Validation

Here we report the RRID for each antibody used. Beta-Actin (Proteintech, 66009-1-lg, RRID: AB_2687938), COXl (Abeam, ab14705, RRID: AB_2084810), COX2 (Abeam, ab110258, RRID: AB_10887758), COXSB (Santa Cruz Biotechnology Inc., sc-374417, RRID: AB_10988066), mS29/DAP3 (Sigma-Aldrich, HPA023687, RRID: AB_1847472) Horseradish peroxidase-conjugated anti-mouse or anti-rabbit lgG's were used a secondary antibodies (Rockland lmmunochemicals, 610-103-121 & 611-1302, RRID: AB_218457 & RRID: AB_219720)

Eukaryotic cell lines

Policy information about cell lines and Sex and Gender in Research

Cell line source(s)

Human HEK293T embryonic kidney cells (CRL-3216, RRID:CVCL-0063) were obtained from ATCC and used to generate all mutant cell lines in this study

A mS29-KO cell line in the HEK293T background was created using a pool of CRISPR/Cas9-mediated knockout cells generated by the genome editing company, Synthego (Synthego Corporation, Redwood City, CA). HEK293T cells were first tested to be negative for mycoplasma. Three guide RNAs targeting exon 5 of the MRPS29 gene (DAP3-202 transcript ID ENST00000368336.10) were selected for high specificity and activity to create premature stop codons through frameshift mutations in the coding region via insertions and/or deletions (lndels). Targeting exon 5 ensured that all MRPS29 transcript variants would be affected. The pool of knockout cells was then generated by electroporation of ribonucleoproteins (RNPs) containing the Cas9 protein and synthetic chemically modified sgRNA (Synthego) into the cells using Synthego's optimized protocol. The editing efficiency was assessed upon recovery, 48 h post-electroporation. Genomic DNA is extracted from a portion of the cells, PCR-amplified, and sequenced using Sanger sequencing. The resulting chromatograms are processed using Synthego Inference of CRISPR Edits software (ice.synthego.com). The pooled MRPS29-KO was then plated into 96-well plates to screen for single clones in-house. Single clone candidates were screened by immunoblotting to determine the steady-state levels of mS29 and the mtDNA-encoded COX2 protein as a surrogate of mitochondrial protein synthesis capacity. Clones that had undetectable mS29 and attenuated COX2 levels were further analyzed by genotyping.

To establish a stable cell line reconstituted with mS29, we cloned the WT MRPS29 gene into a mammalian vector with hygromycin as the selection marker. Two ug of the construct containing MRPS29 was transfected to the HEK293T MRPS29-KO cell line using 5 ul EndoFectin (GeneCopoeia). 72 h post-transfection, the medium was supplemented with 200ug/ml hygromycin for three weeks. The vector used was pCMV6, in which MRPS29 gene expression was placed under the control of an attenuated version of the human cytomegalovirus (CMV) enhancer/promoter (Δ 5) in which a deletion in the promoter sequence eliminates most transcription factor binding sites. We use the same protocol to establish stable cell lines reconstituted with mutant variants of mS29.

Authentication

KO and mutant cell lines were validated by screening single clones via immunoblot against mS29, COX1, and COX2, and subsequent genotyping.

Mycoplasma contamination

Cell lines are regularly tested for mycoplasma contamination using the Sigma "Lookout® Mycoplasma qPCR Detection Kit" and were confirmed mycoplasma free.

Commonly misidentified lines
(See ICLAC register)

No commonly misidentified cell lines were used in this study.
