# Supplementary material for: Mitoribosome structure with cofactors and modifications reveals mechanism of ligand binding and interactions with L1 stalk
Source: Nat Commun. 2024 May 20;15:4272. doi: 10.1038/s41467-024-48163-x (PMC11106087; doi:10.1038/s41467-024-48163-x)
Supplement: Supplementary file 7 — Source Data [file 41467_2024_48163_MOESM7_ESM.zip › Source Data File.docx]

**Original blots**


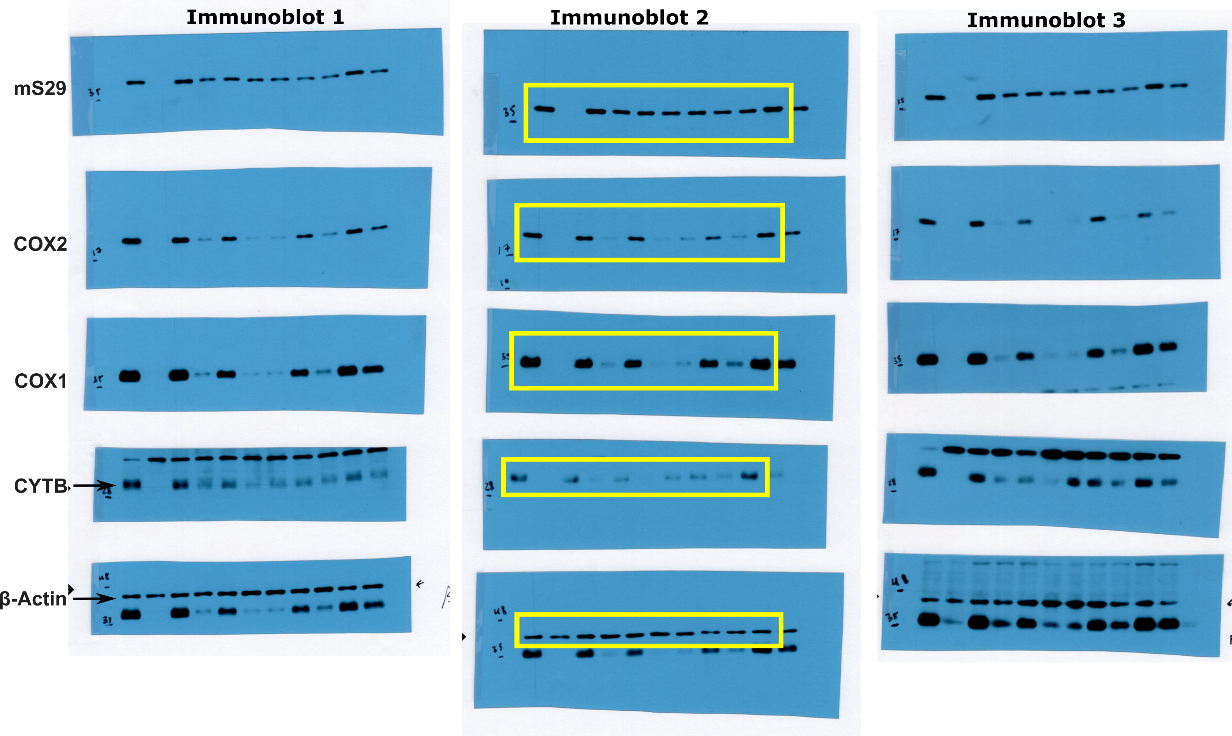


Representative blot shown in Fig.10d

**Original blots**


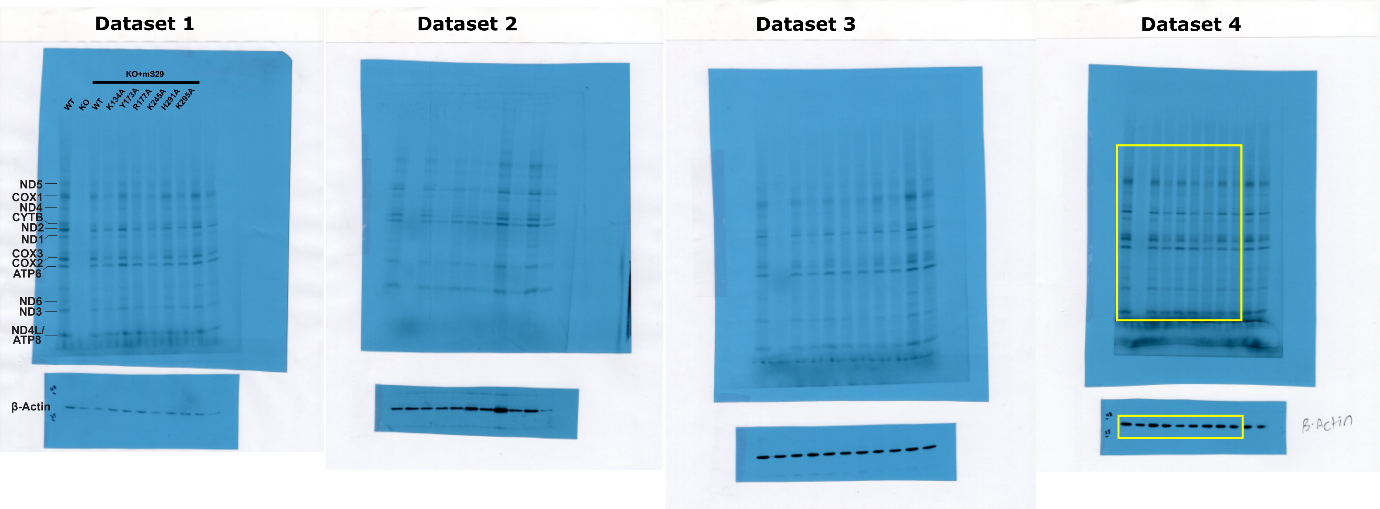


Representative blot shown in Fig.10f
